# The genome sequence of the spotted kaleidoscope jellyfish, *Haliclystus octoradiatus* (James-Clark, 1863)

**DOI:** 10.12688/wellcomeopenres.18669.1

**Published:** 2023-01-05

**Authors:** Mark Blaxter, Darwin Tree of Life Barcoding collective, Wellcome Sanger Institute Tree of Life programme, Wellcome Sanger Institute Scientific Operations: DNA Pipelines collective, Tree of Life Core Informatics collective, Darwin Tree of Life Consortium

**Affiliations:** 1 Wellcome Sanger Institute, Hinxton, Cambridgeshire, UK

**Keywords:** Haliclystus octoradiatus, spotted kaleidoscope jellyfish, genome sequence, chromosomal, Haliclystidae

## Abstract

We present a genome assembly from an individual *Haliclystus octoradiatus* (the spotted kaleidoscope jellyfish; Cnidaria; Staurozoa; Stauromedusae; Haliclystidae). The genome sequence is 262 megabases in span. Most of the assembly (98.3%) is scaffolded into nine (9) chromosomal pseudomolecules. The mitochondrial genome was also assembled and is 18.3 kilobases in length.

## Species taxonomy

Eukaryota; Metazoa; Cnidaria; Staurozoa; Stauromedusae; Myostaurida; Haliclystidae; *Haliclystus*; *Haliclystus octoradiatus* (James-Clark, 1863) (NCBI txid:313498).

## Background

Phylum Cnidaria is an early-branching animal group, with ongoing debate about whether Cnidaria, Ctenophores, Porifera or Placozoa form the earliest-branching clade in Metazoa ( Redmond & McLysaght, 2021). Cnidaria are divided into Anthozoa (corals and anemones), Myxozoa (parasites) and Medusozoa (jellyfish and hydra). *Haliclystus octoradiatus*, the rainbow-stalked jellyfish, is a sessile medusozoan in the class Staurozoa ( Miranda *et al.*, 2016) which lives in the littoral and sublittoral zones attached to the fronds of kelp or other seaweeds. It has a short, seasonal lifecycle with both asexual and sexual stages, and a peak of sexual adult stauromedusoid densities in the summer months ( Miranda *et al.*, 2012). The common name derives from the many different colour morphs of this species and its conspicuous stinging nematocysts, and was invented by David Fenwick following the naming of *Haliclystus auricula* as the “kaleidoscope jellyfish” in a public competition. While *H. octoradiatus* is likely to be the most common *Haliclystus* species in Britain and Ireland, there are relatively few records, mostly from Cornwall and the south-west. The specimen sequenced is the first record from Cumbrae in the Clyde Estuary. The species is not known to be endangered, but comparison of recorded densities through the latter part of the twentieth century in Cornwall suggested a marked decline, perhaps linked to declines in eel-grass and other inshore algal ecosystems ( Hiscock *et al.*, 2011).

By sequencing the genome of *H. octoradiatus* we hope to both offer additional data that may be of utility in deep and local phylogenetic analyses of Staurozoa and Cnidaria, and also may form the foundations of a toolkit for the deployment of eDNA and other approaches to monitoring otherwise inaccessible biodiversity.

### Genome sequence report

**Figure 1.**
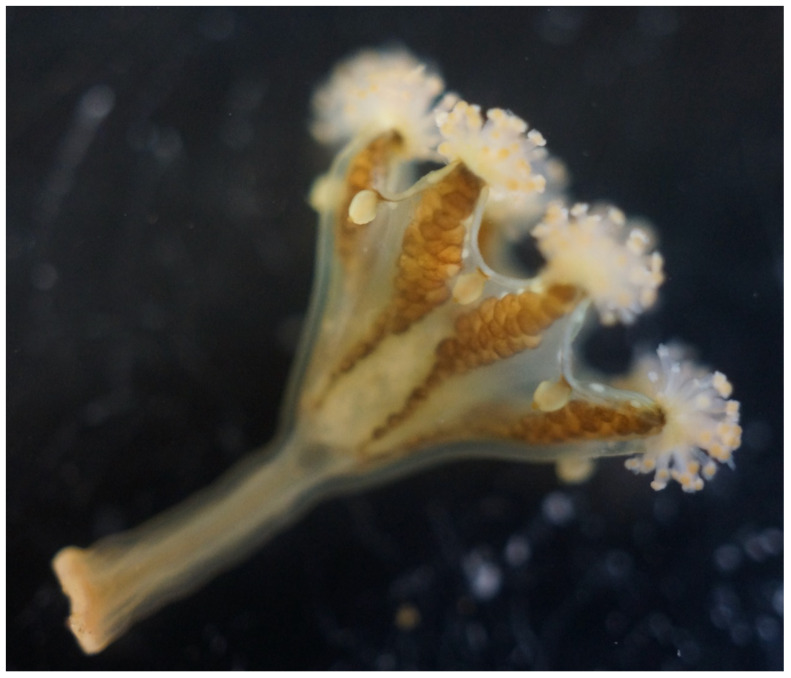
*Haliclystus octoradiatus* (sequenced specimen; 2 cm long). White Bay, Great Cumbrae, Ayrshire. 23 August 2020. Mark Blaxter.

The genome was sequenced from a single *H. octoradiatus* ( Figure 1) collected from Great Cumbrae, Scotland (55.79, – 4.91). A total of 68-fold coverage in Pacific Biosciences single-molecule HiFi long reads and 147-fold coverage in 10X Genomics read clouds was generated. Primary assembly contigs were scaffolded with chromosome conformation Hi-C data. Manual assembly curation corrected 260 missing or mis-joins and removed 33 haplotypic duplications, reducing the assembly length by 1.03% and the scaffold number by 80.24%, and increasing the scaffold N50 by 91.38%.

**Figure 2.**
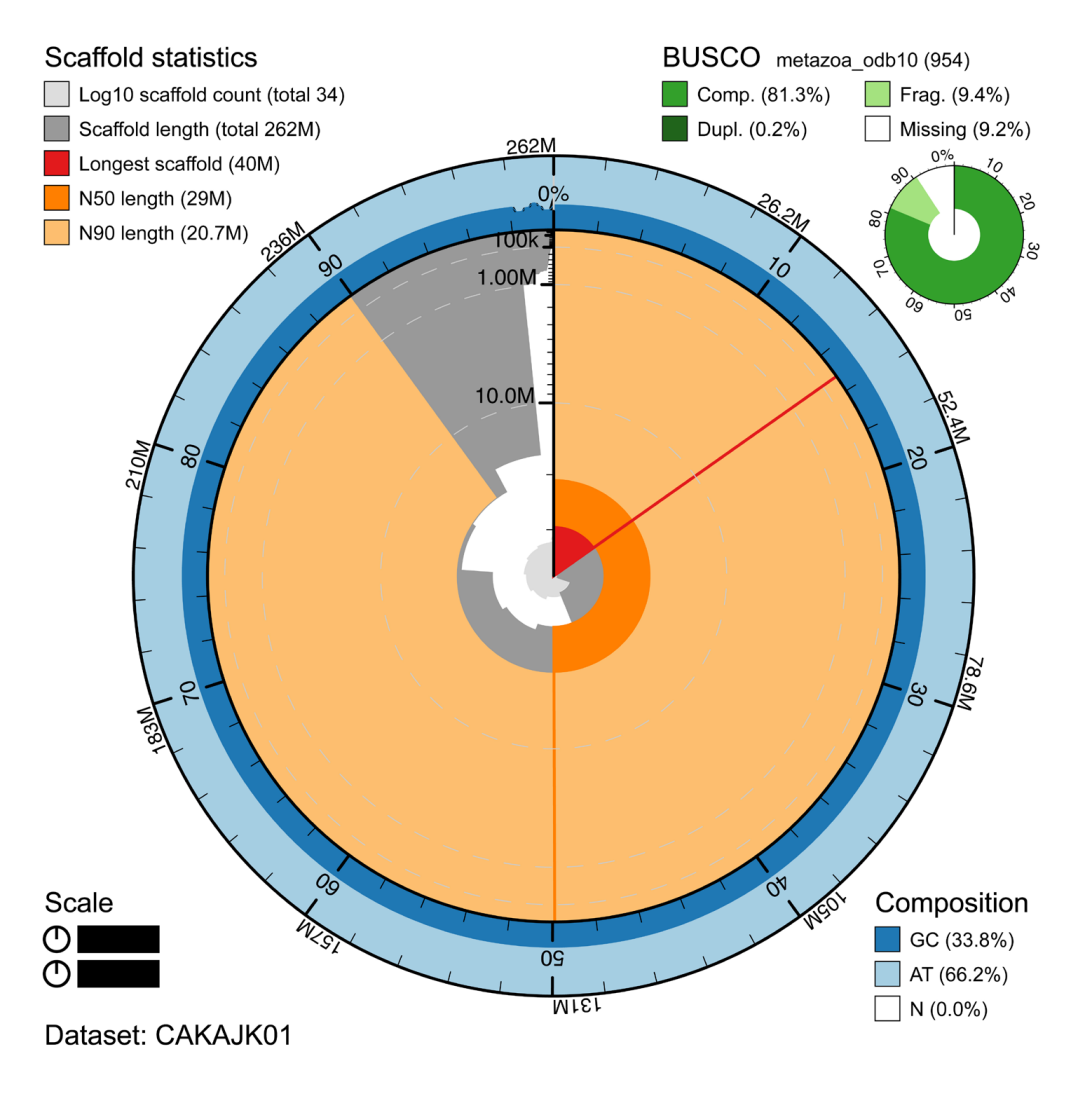
Genome assembly of *Haliclystus octoradiatus* (jrHalOcto1.1): metrics. The BlobToolKit Snailplot shows N50 metrics and BUSCO gene completeness. The main plot is divided into 1,000 size-ordered bins around the circumference with each bin representing 0.1% of the 261,898,343 bp assembly. The distribution of chromosome lengths is shown in dark grey with the plot radius scaled to the longest chromosome present in the assembly (39,977,743 bp, shown in red). Orange and pale-orange arcs show the N50 and N90 chromosome lengths (28,991,210 and 20,733,292 bp), respectively. The pale grey spiral shows the cumulative chromosome count on a log scale with white scale lines showing successive orders of magnitude. The blue and pale-blue area around the outside of the plot shows the distribution of GC, AT and N percentages in the same bins as the inner plot. A summary of complete, fragmented, duplicated and missing BUSCO genes in the metazoa_odb10 set is shown in the top right. An interactive version of this figure is available at https://blobtoolkit.genomehubs.org/view/jrHalOcto1.1/dataset/CAKAJK01/snail.

The final assembly has a total length of 262 Mb in 33 sequence scaffolds with a scaffold N50 of 29 Mb ( Table 1). Most (98.3%) of the assembly sequence was assigned to 9 chromosomal-scale scaffolds. Chromosome-scale scaffolds confirmed by the Hi-C data are named in order of size ( Figure 2– Figure 5; Table 2). While not fully phased, the assembly deposited is of one haplotype. Contigs corresponding to the second haplotype have also been deposited. The mitochondrial genome was assembled also.

**Table 1.** Genome data for *Haliclystus octoradiatus* (jrHalOcto1.1).

Project accession data		
Assembly identifier	jrHalOcto1.1	
Species	*Haliclystus octoradiatus*	
Specimen	jrHalOcto1	
NCBI taxonomy ID	313498	
BioProject	PRJEB45172	
BioSample ID	SAMEA7522858	
Isolate information	mid-body tissue	
Assembly metrics *		*Benchmark*
Consensus quality (QV)	56.6	*≥ 50*
*k*-mer completeness	99.99%	*≥ 95%*
BUSCO **	C:81.3%[S:81.1%,D:0.2%], F:9.4%,M:9.2%,n:954	*C ≥ 95%*
Percentage of assembly mapped to chromosomes	98.3%	*≥ 95%*
Sex chromosomes	Not applicable	*localised homologous pairs*
Organelles	mitochondrial genome 18.3 kb	*complete single alleles*
Raw data accessions		
PacificBiosciences SEQUEL II	ERR6897437, ERR6897438	
10X Genomics Illumina	ERR6745734– ERR6745737	
Hi-C Illumina	ERR6745733	
PolyA RNA-Seq Illumina	ERR9434978	
Genome assembly		
Assembly accession	GCA_916610825.1	
*Accession of alternate haplotype*	GCA_916610655.1	
Span (Mb)	262	
Number of contigs	337	
Contig N50 length (Mb)	1.5	
Number of scaffolds	33	
Scaffold N50 length (Mb)	29	
Longest scaffold (Mb)	40	

* Assembly metric benchmarks are adapted from column VGP-2020 of “Table 1: Proposed standards and metrics for defining genome assembly quality” from ( Rhie *et al.*, 2021).

** BUSCO scores based on the metazoa_odb10 BUSCO set using v5.1.2. C = complete [S = single copy, D = duplicated], F = fragmented, M = missing, n = number of orthologues in comparison. A full set of BUSCO scores is available at https://blobtoolkit.genomehubs.org/view/jrHalOcto1.1/dataset/CAKAJK01/busco.

**Figure 3.**
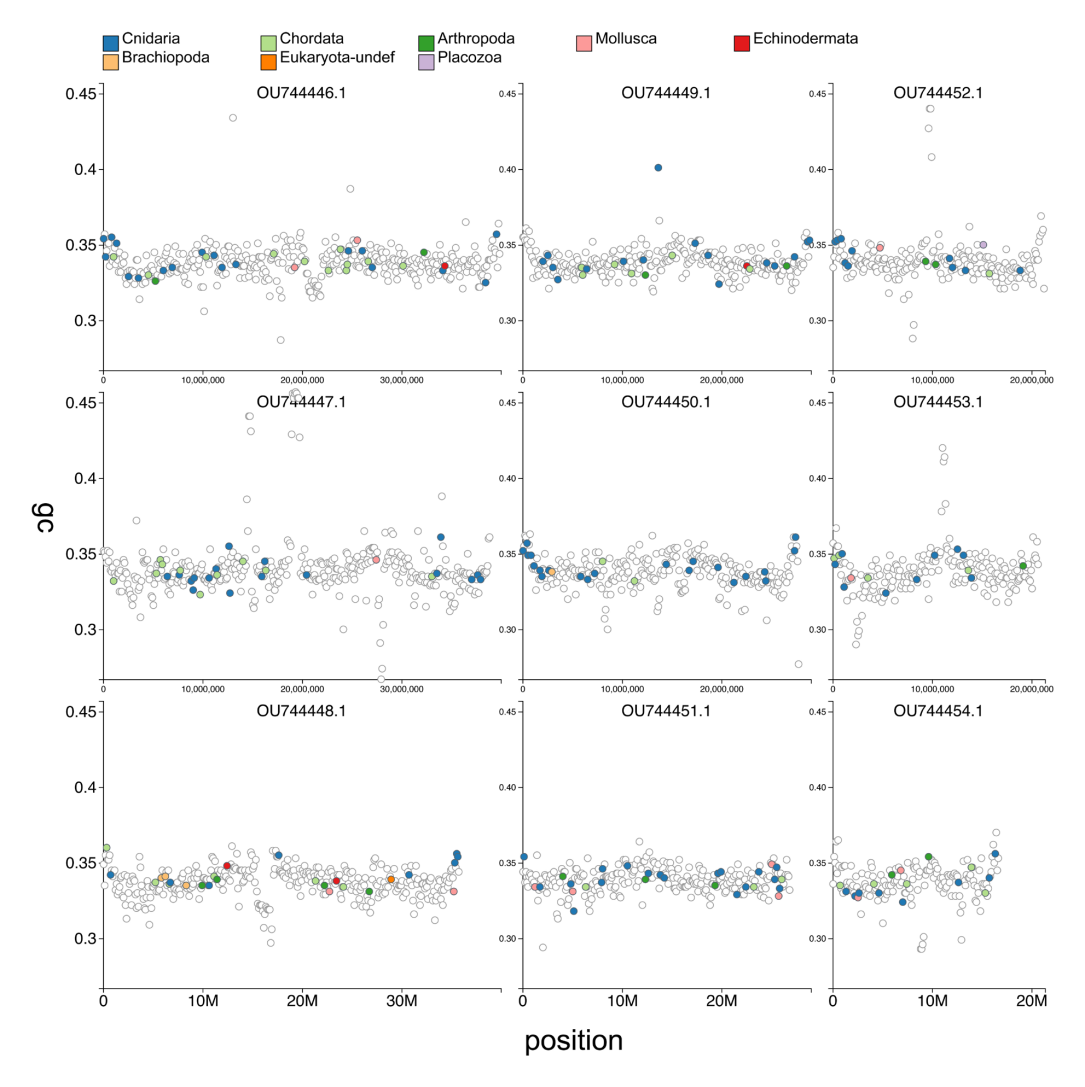
Genome assembly of *Haliclystus octoradiatus* jrHalOcto1.1): GC coverage on the nine chromosomal molecules. BlobToolKit GC-position plot. CG coverage of each chromosome (indicated by its INSDC accession OU744446.1–OU744454.1) plotted in non-overlapping bins of 100 kb. Points are coloured blue if the region contains a match to a Cnidaria BUSCO locus. Other colours indicate regions with matches to other phyla. An interactive version of this figure is available at https://blobtoolkit.genomehubs.org/view/Cnidaria/dataset/CAKAJK01/blob?plotShape=grid&position--Active=true&xField=position&yField=gc&windowSize=100000&length--Min=1220000.

**Figure 4.**
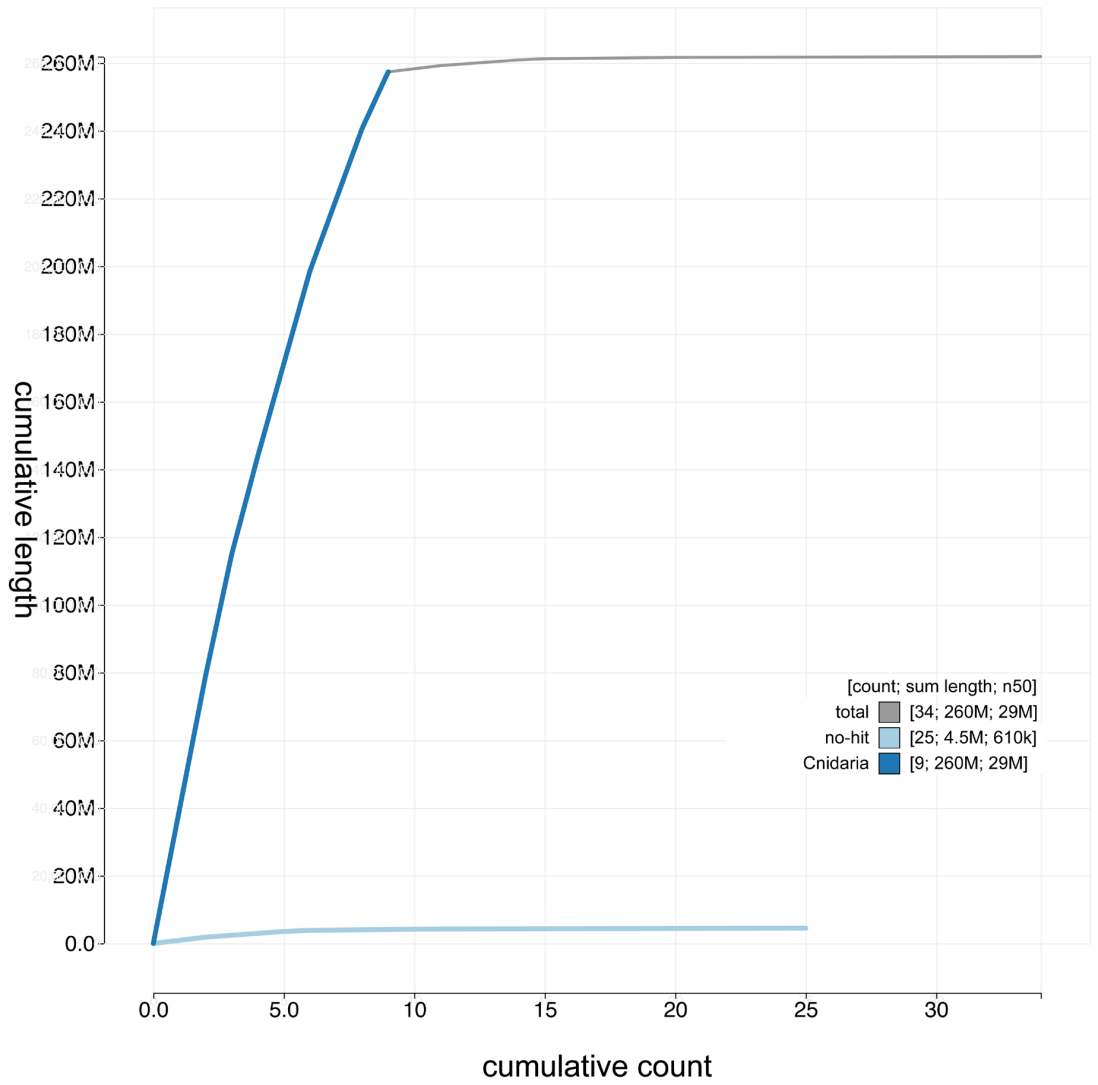
Genome assembly of *Haliclystus octoradiatus* (jrHalOcto1.1): cumulative sequence. BlobToolKit cumulative sequence plot. The grey line shows cumulative length for all chromosomes. Coloured lines show cumulative lengths of chromosomes assigned to each phylum using the buscogenes taxrule. An interactive version of this figure is available at https://blobtoolkit.genomehubs.org/view/jrHalOcto1.1/dataset/CAKAJK01/cumulative.

**Figure 5.**
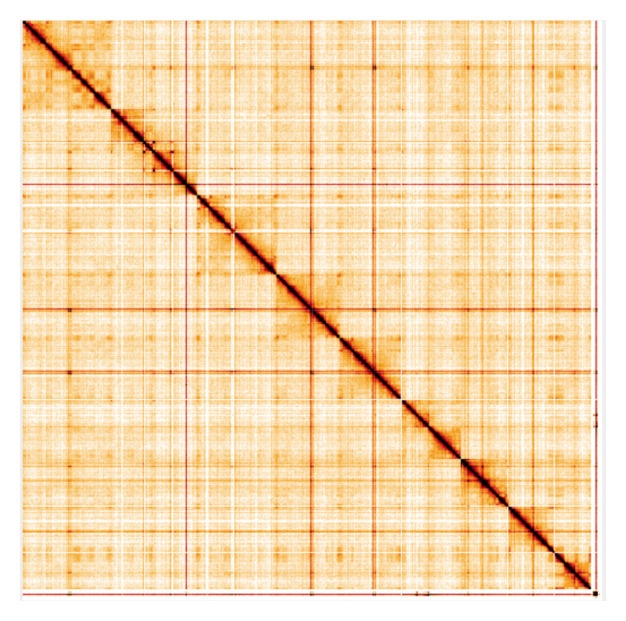
Genome assembly of *Haliclystus octoradiatus* (jrHalOcto1.1): Hi-C contact map. Hi-C contact map of the jrHalOcto1.1 assembly, visualised using HiGlass. Chromosomes are shown in order of size from left to right and top to bottom. An interactive version of this figure may be viewed at https://genome-note-higlass.tol.sanger.ac.uk/l/?d=etAxpojERhyq9MIacE4cPQ.

**Table 2.** Chromosomal pseudomolecules in the genome assembly of *Haliclystus octoradiatus* (jrHalOcto1).

INSDC accession	Chromosome	Size (Mb)	GC%
OU744446.1	1	39.98	33.8
OU744447.1	2	38.98	34.1
OU744448.1	3	35.87	33.7
OU744449.1	4	28.99	33.9
OU744450.1	5	27.81	33.8
OU744451.1	6	26.96	33.9
OU744452.1	7	21.35	33.9
OU744453.1	8	20.73	33.8
OU744454.1	9	16.71	33.7
OU744455.1	MT	0.02	38.3
-	-	4.49	30.5

The assembly has a BUSCO v5.1.2 ( Manni *et al.*, 2021) completeness of 81.5% (single 81.3%, duplicated 0.2%) using the OrthoDB-v10 metazoa reference set. BUSCO loci identified as fragmented accounted for a further 9.4% of loci tested. This low BUSCO score may be due to low conservation of orthologues between *H. octoradiatus* and the metazoan species in the reference set, or underperformance of the BUSCO gene finder given the particular gene structures in this species. The assembly is validated by the other assembly quality metrics ( *k*-mer completeness 99.9%, consensus quality (QV) 56.6) shown in Table 1.

## Methods

### Sample acquisition and nucleic acid extraction

An individual *H. octoradiatus* (jrHalOcto1) was collected from White Bay in Great Cumbrae, Scotland (latitude 55.79, longitude –4.91). The sample was caught by hand from a rockpool and identified by Mark Blaxter (Wellcome Sanger Institute). The specimen was identified by its morphology, using a dichotomous key ( Hayward & Ryland, 2017). The specimen was preserved and shipped on dry ice.

DNA was extracted at the Tree of Life laboratory, Wellcome Sanger Institute. The jrHalOcto1 sample was weighed and dissected on dry ice with tissue set aside for Hi-C sequencing. The mid-body tissue was disrupted using a Nippi Powermasher fitted with a BioMasher pestle *.* High molecular weight (HMW) DNA was extracted using the Qiagen MagAttract HMW DNA extraction kit. Low molecular weight DNA was removed from a 20-ng aliquot of extracted DNA using 0.8X AMpure XP purification kit prior to 10X Chromium sequencing and a minimum of 50 ng DNA was submitted for 10X sequencing. HMW DNA was sheared into an average fragment size of 12–20 kb in a Megaruptor 3 system with speed setting 30. Sheared DNA was purified by solid-phase reversible immobilisation using AMPure PB beads with a 1.8X ratio of beads to sample to remove the shorter fragments and concentrate the DNA sample. The concentration of the sheared and purified DNA was assessed using a Nanodrop spectrophotometer and Qubit Fluorometer and Qubit dsDNA High Sensitivity Assay kit. Fragment size distribution was evaluated by running the sample on the FemtoPulse system.

RNA was extracted from mid-body tissue of jrHalOcto1 in the Tree of Life Laboratory using TRIzol, according to the manufacturer’s instructions. RNA was then eluted in 50 μl RNAse-free water and its concentration assessed using a Nanodrop spectrophotometer and Qubit Fluorometer using the Qubit RNA Broad-Range (BR) Assay kit. Analysis of the integrity of the RNA was done using Agilent RNA 6000 Pico Kit and Eukaryotic Total RNA assay.

### Sequencing

Pacific Biosciences HiFi circular consensus and 10X Genomics read cloud DNA sequencing libraries were constructed according to the manufacturers’ instructions. Poly(A) RNA-Seq libraries were constructed using the NEB Ultra II RNA Library Prep kit. DNA and RNA sequencing was performed by the Scientific Operations core at the WSI on Pacific Biosciences SEQUEL II (HiFi), Illumina HiSeq 4000 (RNA-Seq) and Illumina NovaSeq 6000 (10X) instruments. Hi-C data were also generated from mid-body tissue of jrHalOcto1 using the Arimav2 kit and sequenced on the HiSeq X Ten instrument.

### Genome assembly

Assembly was carried out with HiCanu ( Nurk *et al.*, 2020) and haplotypic duplication was identified and removed with purge_dups ( Guan *et al.*, 2020). One round of polishing was performed by aligning 10X Genomics read data to the assembly with Long Ranger ALIGN, calling variants with freebayes ( Garrison & Marth, 2012). The assembly was then scaffolded with Hi-C data ( Rao *et al.*, 2014) using SALSA2 ( Ghurye *et al.*, 2019). The assembly was checked for contamination and corrected using the gEVAL system ( Chow *et al.*, 2016) as described previously ( Howe *et al.*, 2021). Manual curation was performed using gEVAL, HiGlass ( Kerpedjiev *et al.*, 2018) and Pretext ( Harry, 2022). The mitochondrial genome was assembled using MitoHiFi ( Uliano-Silva *et al.*, 2021), which performed annotation using MitoFinder ( Allio *et al.*, 2020). The genome was analysed and BUSCO scores were generated within the BlobToolKit environment ( Challis *et al.*, 2020). Table 3 contains a list of all software tool versions used, where appropriate.

**Table 3.** Software tools and versions used.

Software tool	Version	Source
BlobToolKit	2.6.2	Challis *et al.*, 2020
freebayes	1.3.1-17- gaa2ace8	Garrison & Marth, 2012
HiCanu	0.15.3	Nurk *et al.*, 2020
HiGlass	1.11.6	Kerpedjiev *et al.*, 2018
Long Ranger ALIGN	2.2.2	https://support.10xgenomics.com/ genome-exome/software/pipelines/ latest/advanced/other-pipelines
MitoHiFi	1.0	Uliano-Silva *et al.*, 2021
PretextView	0.2.x	Harry, 2022
purge_dups	1.2.3	Guan *et al.*, 2020
SALSA2	2.2	Ghurye *et al.*, 2019

## Data Availability

European Nucleotide Archive: *Haliclystus octoradiatus* (spotted kaleidoscope jellyfish). Accession number PRJEB45172; https://identifiers.org/ena.embl/PRJEB45172 ( Wellcome Sanger Institute, 2022) The genome sequence is released openly for reuse. The *Haliclystus octoradiatus* genome sequencing initiative is part of the Darwin Tree of Life (DToL) project. All raw sequence data and the assembly have been deposited in INSDC databases. The genome will be annotated using available RNA-Seq data and presented through the Ensembl pipeline at the European Bioinformatics Institute. Raw data and assembly accession identifiers are reported in Table 1. Members of the Darwin Tree of Life Barcoding collective are listed here: https://doi.org/10.5281/zenodo.4893703. Members of the Wellcome Sanger Institute Tree of Life programme are listed here: https://doi.org/10.5281/zenodo.4783585. Members of Wellcome Sanger Institute Scientific Operations: DNA Pipelines collective are listed here: https://doi.org/10.5281/zenodo.4790455. Members of the Tree of Life Core Informatics collective are listed here: https://doi.org/10.5281/zenodo.5013541. Members of the Darwin Tree of Life Consortium are listed here: https://doi.org/10.5281/zenodo.4783558.
