# Supplementary material for: The genome sequence of the spotted kaleidoscope jellyfish, Haliclystus octoradiatus (James-Clark, 1863)
Source: Wellcome Open Res. 2023 Jan 5;8:6. [Version 1] doi: 10.12688/wellcomeopenres.18669.1 (PMC9975415; doi:10.12688/wellcomeopenres.18669.1)
Supplement: Peer review history

# Peer review: Reviewer response for version 1

**DOI:** 10.21956/wellcomeopenres.20703.r53901

**Published:** 2023-01-26

**Authors:** Robert E. Steele

**Affiliations:** 1 Department of Biological Chemistry, University of California Irvine, Irvine, CA, USA

Staurozoa is a much under-studied class of the phylum Cnidaria. The class was first established in 2004 by Marques and Collins, and its position in the cnidarian tree was not firmly established until 2018, also by Collins and collaborators. Thus we still have much to learn about this class. One other staurozoan genome assembly has been generated, from *Calvadosia cruxmelitensis*. It was published in 2019, and is highly fragmented, with an N50 of only 16 kb. Thus this current chromosome scale assembly from the staurozoan *Haliclystus octoradiatus* is a very welcome addition. However, I am surprised that the authors do not cite the *Calvadosia* paper, which was published in Peer J. by Ohdera et al.  This needs to be done. The finding that *H. octoradiatus* has only 9 chromosomes is quite surprising, given that cnidarians typically have 15 chromosomes. It will be of interest to see what is learned about chromosome dynamics within Cnidaria when the *H. octoradiatus* karyotype is compared to chromosome scale assemblies from other cnidarians. In addition, the authors provide an assembly of the mitochondrial genome, which is an important resource. The data and analyses provided by this study are of very high quality and will be valuable resources for the metazoan genomics research community.

Are sufficient details of methods and materials provided to allow replication by others?

Yes

Is the rationale for creating the dataset(s) clearly described?

Yes

Are the datasets clearly presented in a useable and accessible format?

Yes

Are the protocols appropriate and is the work technically sound?

Yes

Reviewer Expertise:

Cnidarian biology.  Metazoan genomics.

I confirm that I have read this submission and believe that I have an appropriate level of expertise to confirm that it is of an acceptable scientific standard.

# Peer review: Reviewer response for version 1

**DOI:** 10.21956/wellcomeopenres.20703.r53900

**Published:** 2023-01-26

**Authors:** Richard R. Copley

**Affiliations:** 1 Laboratoire de Biologie du Développement de Villefranche-sur-mer (LBDV), CNRS, Sorbonne Université, Villefranche-sur-mer, France

Blaxter and colleagues report the genome sequence of staurozoan cnidarian. Staurozoa are sometimes called 'stalked-jellyfish'. They are significant in that they fall with the cnidarian clade of Medusozoa, but without actually producing a free living medusa form - in this they resemble the Anthozoa. A possible phylogenetic position as sister to a clade of Scyphozoa and Cubozoa ^1^ raises the interesting possibility that medusae have evolved independently in Hydrozoa. Contrary to the first sentence of this article, Cnidaria as a whole are unlikely to be anything other than sister to the Bilateria, possibly with Ctenophores or Placozoans, as per the Redmond and McLysaght article cited (cf. Redmond & McLysaght Figure 5).

 The genome sequence appears excellent from the summary statistics, although possibly the BUSCO numbers are a little lower than one might expect. This can be further followed up when gene predictions are available. I was able to find a few key genes we would expect in Staurozoa with little trouble. All in all, this is a valuable contribution to cnidarian genomes.

Are sufficient details of methods and materials provided to allow replication by others?

Yes

Is the rationale for creating the dataset(s) clearly described?

Yes

Are the datasets clearly presented in a useable and accessible format?

Yes

Are the protocols appropriate and is the work technically sound?

Yes

Reviewer Expertise:

cnidarian genomics; animal evolution;

I confirm that I have read this submission and believe that I have an appropriate level of expertise to confirm that it is of an acceptable scientific standard.

## References

[1] : Phylogenomics provides a robust topology of the major cnidarian lineages and insights on the origins of key organismal traits. *BMC Evolutionary Biology* .2018;18(1) : 10.1186/s12862-018-1142-0 10.1186/s12862-018-1142-0 29390964
